# The diamond–silicon carbide composite Skeleton^®^ as a promising material for substrates of intense X-ray beam optics

**DOI:** 10.1107/S1600577524006088

**Published:** 2024-08-06

**Authors:** Alexey E. Pestov, Aleksei Ya. Lopatin, Petr V. Volkov, Maria V. Zorina, Andrei Yu. Lukyanov, Ilya V. Malyshev, Mikhail S. Mikhailenko, Mikhail N. Toropov, Daniil A. Semikov, Aleksei K. Chernyshev, Nikolay I. Chkhalo, Pavel A. Yunin, Egor I. Glushkov, Sergey K. Gordeev, Svetlana B. Korchagina

**Affiliations:** a Institute for Physics of Microstructures RAS Nizhny Novgorod603087 Russian Federation; b CSRI of Materials, St Petersburg191014, Russian Federation; Bhabha Atomic Research Centre, India

**Keywords:** carbon composites, X-ray optics, synchrotron mirrors, roughness, thermophysical characteristics

## Abstract

The paper considers the possibility of using the diamond-silicon carbide composite Skeleton^®^ with a technological coating of polycrystalline silicon as a substrate for X-ray mirrors used with powerful synchrotron radiation sources (third+ and fourth generation). Samples were studied after polishing to provide the following surface parameters: root-mean-square flatness ≃ 50 nm, micro-roughness on the frame 2 µm × 2 µm σ ≃ 0.15 nm. The heat capacity, thermal conductivity and coefficient of linear thermal expansion were investigated. For comparison, a monocrystalline silicon sample was studied under the same conditions using the same methods. The value of the coefficient of linear thermal expansion turned out to be higher than that of monocrystalline silicon and amounted to 4.3 × 10^−6^ K^−1^, and the values of thermal conductivity (5.0 W cm^−1^ K^−1^) and heat capacity (1.2 J K^−1^ g^−1^) also exceeded the values for Si. Thermally induced deformations of both Skeleton^®^ and monocrystalline silicon samples under irradiation with a CO_2_ laser beam have also been experimentally studied. Taking into account the obtained thermophysical constants, the calculation of thermally induced deformation under irradiation with hard (20 keV) X-rays showed almost three times less deformation of the Skeleton^®^ sample than of the monocrystalline silicon sample.

## Abstract 2

A new material for substrates of optical elements used under ultra-powerful X-ray beams is described – the diamond-silicon carbide composite Skeleton^®^. The composition and structure of the material, its surface roughness with a technological coating of polycrystalline silicon and its thermophysical characteristics are studied.

## 1. Introduction

With the development of powerful synchrotron radiation (SR) sources and of free-electron lasers, the problem of manufacturing precise optical elements for X-rays that are resistant to large (up to several kilowatts) amounts of radiation and thermal loads has become acute. Currently, monocrystalline silicon is primarily considered as a substrate material for mirrors operating under powerful radiation beams (Belure *et al.*, 2020 ▸; Assoufid & Graafsma, 2017 ▸; Wang *et al.*, 2022 ▸). Other materials, including Zerodur, SiC and metals (copper, aluminium and beryllium) (Belure *et al.*, 2019 ▸; Khounsary *et al.*, 2002 ▸; Chkhalo *et al.*, 2019 ▸; DiGennaro *et al.*, 1988 ▸), are inferior to silicon in either cost, polishability or thermophysical characteristics. Since silicon’s thermal conductivity increases about tenfold when the temperature changes from ambient to cryogenic, liquid nitro­gen cooling of the substrate is always used for efficient heat transfer. A considerable disadvantage of cryogenic cooling of X-ray mirrors is the overall complexity of the design. Besides that, increased mirror vibration caused by boiling coolant can negatively affect the spatial stability of the reflected beam. These are all reasons to continue the search for an alternative substrate material with high thermal conductivity at room temperature, which can be effectively combined with a simple and reliable water cooling system. Monocrystalline diamond has the best characteristics in this sense but there are difficulties in obtaining it with dimensions of tens of centimetres (Shvyd’ko *et al.*, 2021 ▸).

As an alternative to monocrystalline silicon, we offer Skeleton^®^, a diamond–silicon carbide composite (DSCC) (Kataev *et al.*, 2011 ▸). The microstructure of the DSCC Skeleton^®^ is formed by diamond grains bound into a single composite with a silicon carbide matrix. The material is inferior only to monocrystalline diamond in its physical-mechanical properties (Kataev *et al.*, 2011 ▸); however, unlike diamond, it allows the formation of samples of almost arbitrary size and shape (the ‘net-shape’ technology – chemical reactions within the volume of the sample) and thus allows manufacture of an extended surface to the reverse side of the substrate to increase heat transfer for liquid cooling. The cost-effective nature of this material should also be noted.

The high degree of hardness and rigidity of the Skeleton^®^ composite introduces extreme complications for direct mechanical processing, including grinding and polishing. To solve this problem, a thin (0.5 mm) technological coating of polycrystalline silicon was deposited on the studied samples of Skeleton^®^ using the chemical vapour deposition method. Such a coating allows polishing, yet, due to its small thickness, it should not noticeably reduce the thermal characteristics.

The use of the DSCC Skeleton^®^ to generate substrates for X-ray mirrors operating under powerful synchrotron radiation beams makes it possible to switch from the treatment of difficult-to-process monocrystalline materials to the use of traditional technology for finishing, shaping and polishing as used on substrates of fused silica, Zerodur, sitall *etc*. This approach combines lapping and deep grinding/polishing, as well as finishing ion-beam correction of local shape errors (Chkhalo *et al.*, 2020 ▸). Based on the requirements for the surface of X-ray mirror substrates, at the manufacturing stage it is necessary to achieve subnanometre precision in shape and roughness. To preserve the diffraction quality of wavefronts at the reflection of X-ray radiation with a wavelength λ from a mirror, the permissible root-mean-square (r.m.s.) error in the shape of the mirror, according to the Marechal criterion, should satisfy the inequality r.m.s. ≤ λ/14 (Born & Wolf, 1999 ▸). Taking into account the grazing incidence of X-ray radiation at a grazing angle θ, this relation can be rewritten as
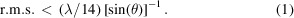
If we substitute the characteristic values of the parameters into (1), for example from Morawe *et al.* (2013 ▸) (mirror W/B_4_C, λ = 0.1 nm, θ = 1.4°), we obtain the permissible shape error at the level of 0.3 nm.

To ensure high reflectivity of multilayer mirrors, the micro-roughness of the substrates must be less than the interlayer roughness, which, in the case of W/B_4_C, is about 0.2 nm (Andreev *et al.*, 2003 ▸). In the case of mirrors with a single-layer reflective coating, these requirements are reduced by three to four times but they remain at the level of 1 nm.

This paper investigates the thermophysical properties of the DSCC Skeleton^®^ material with a technological coating of polycrystalline silicon, and the possibility of using standard polishing techniques. For determining the reliability of the measurements of the thermophysical characteristics, a sample of monocrystalline silicon was also studied under the same conditions and using the same methods.

## 2. Investigation of the DSCC Skeleton^®^

### 2.1. Microstructure of Skeleton^®^

**Figure 1:**
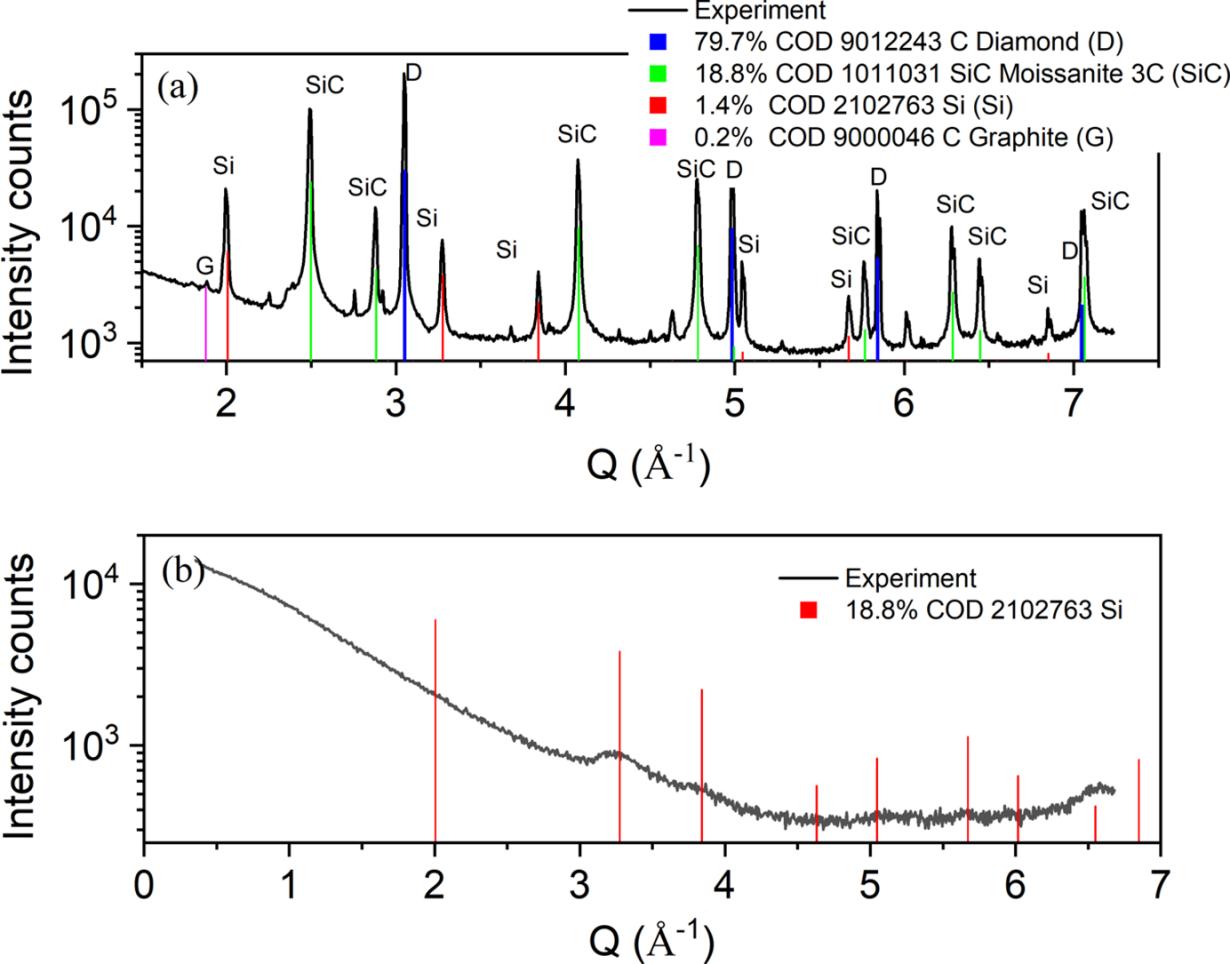
Diffractograms of sample SK2 (λ = 0.154 nm), (*a*) from the reverse (DSCC Skeleton^®^) side and (*b*) from the front (polished Si) side.

Two plates (SK1 and SK2) with a diameter of 40 mm and a thickness of 4.4 mm (4 mm of Skeleton^®^ + 0.4 mm of technological silicon) were used as experimental samples. The structural parameters of the material and the technological coating were investigated using X-ray diffraction (XRD) and Raman spectroscopy. Wide-angle X-ray diffraction is an integral method, allowing the composition of the sample to be determined using XRD data. The studies were carried out on a Bruker D8 Discover X-ray diffractometer (Cu *K*α, λ = 0.154 nm) and signals from the front and reverse sides of the sample were recorded. Fig. 1 ▸ shows the measured diffractograms in momentum-transfer (*Q*) scale, 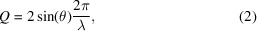
where θ is the grazing angle and λ is the wavelength. The solid line represents the experimental X-ray diffraction curves, while the vertical lines represent the positions of peaks corresponding to different phases of the material, as given by the Crystallography Open Database (COD) (https://www.crystallography.net/cod). Thus, according to the XRD data, the phase composition of the DSCC Skeleton^®^ (the reverse side of the sample) is: C (diamond) 79.7%, SiC 18.8%, Si 1.4% and C (graphite) 0.2%, while on the technological coating (the front side of the sample) there is only nanocrystalline silicon (nanocrystallite sizes are about 2 nm).

### 2.2. Sample surface investigation

The surface roughness was measured using an Ntegra Prima (NT-MDT) atomic force microscope (AFM)-based stand (Chkhalo *et al.*, 2015 ▸). The method of estimating the value of the r.m.s. roughness was based on the approach described by Barysheva *et al.* (2019 ▸), which consists of restoring the PSD (power spectral density) function of the roughness from frames obtained with an AFM. In the present work, frames ranging from 2 µm × 2 µm to 40 µm × 40 µm were taken. The PSD function was determined using formula (3) and is essentially a decomposition of the roughness over the frequencies of the spatial spectrum (Ulmeanu *et al.*, 2000 ▸),
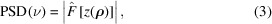
where 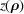 is the height of the surface at the point given by the radius vector 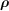 and 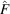 is the Fourier transform. If *L* is the linear size of the scanning area (AFM frame) and *N* is the number of points (pixels), then the modulus of the spatial frequency vector in which the PSD function is calculated lies in the range from ν_min_ = 1/*L* to ν_max_ = *N*/2*L*.

For a quantitative description of surface irregularities, the concept of ‘effective roughness’ is used. Effective roughness is an integral of the PSD function in a certain range of spatial frequencies,
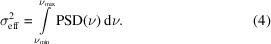
In our case, the spatial frequency range was ν ∈ [2.5 × 10^−2^ to 6.4 × 10^1^ µm^−1^]. This interval covers irregularities with lateral dimensions ranging from 40 µm to 15 nm, which affect both the imaging properties of the optical element and the reflective characteristics of multilayer X-ray mirrors.

Surface flatness measurements were studied using a ZYGO Verifire 4 laser interferometer (ZYGO Corporation). According to the measurements, the following surface parameters were calculated: PV (peak-to-valley – the span of heights on the surface) and r.m.s. (the standard deviation of the surface from the plane).

**Figure 2:**
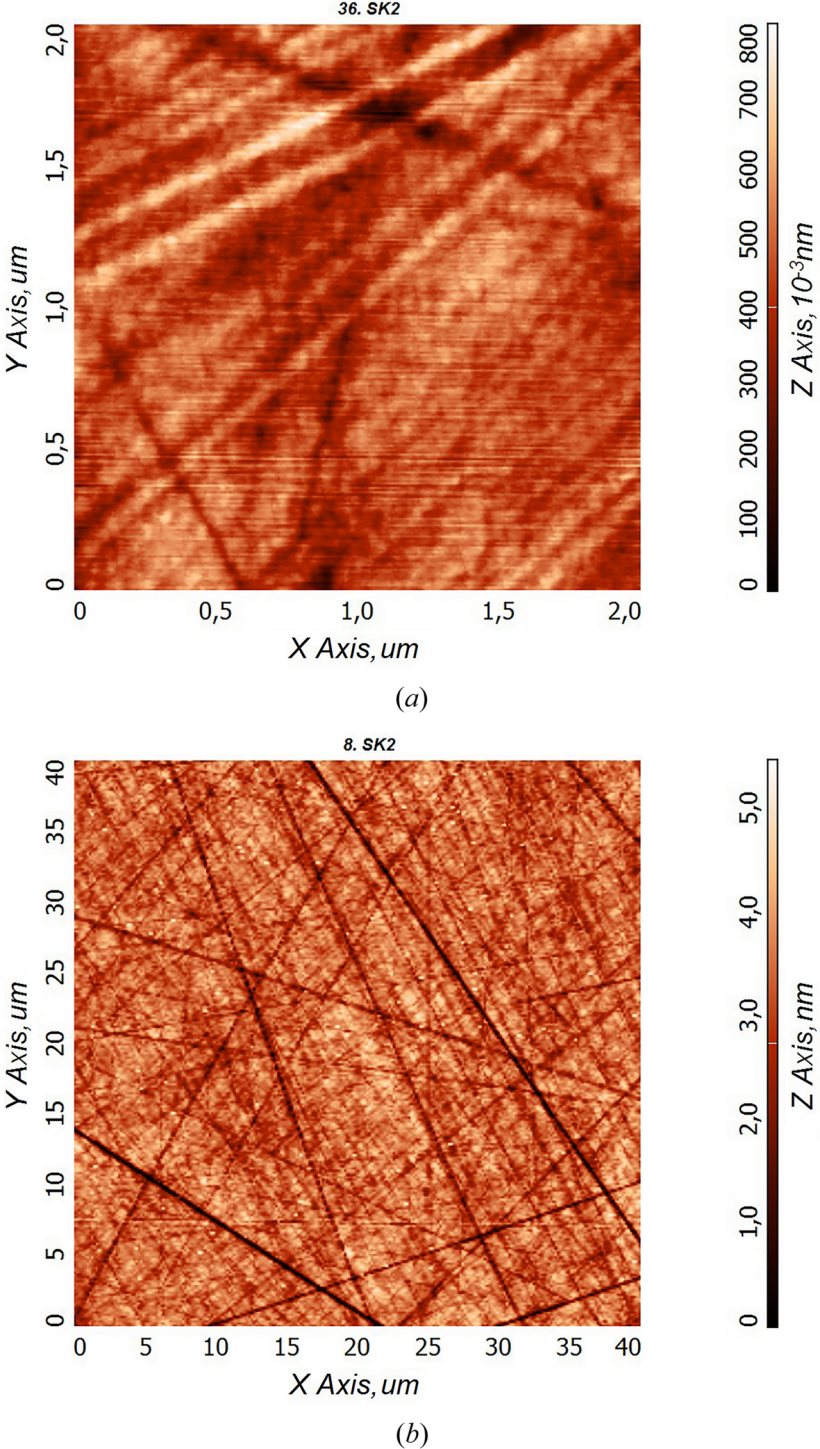
AFM frames of the surface of sample SK2. (*a*) Frame 2 µm × 2 µm, σ_2×2_ = 0.15 nm. (*b*) Frame 40 µm × 40 µm, σ_40×40_ = 0.7 nm.

The measured samples showed similar parameters, in terms of both the mean-square roughness of the surface and the flatness. Fig. 2 ▸ shows typical AFM surface frames for the sample SK2.

**Figure 3:**
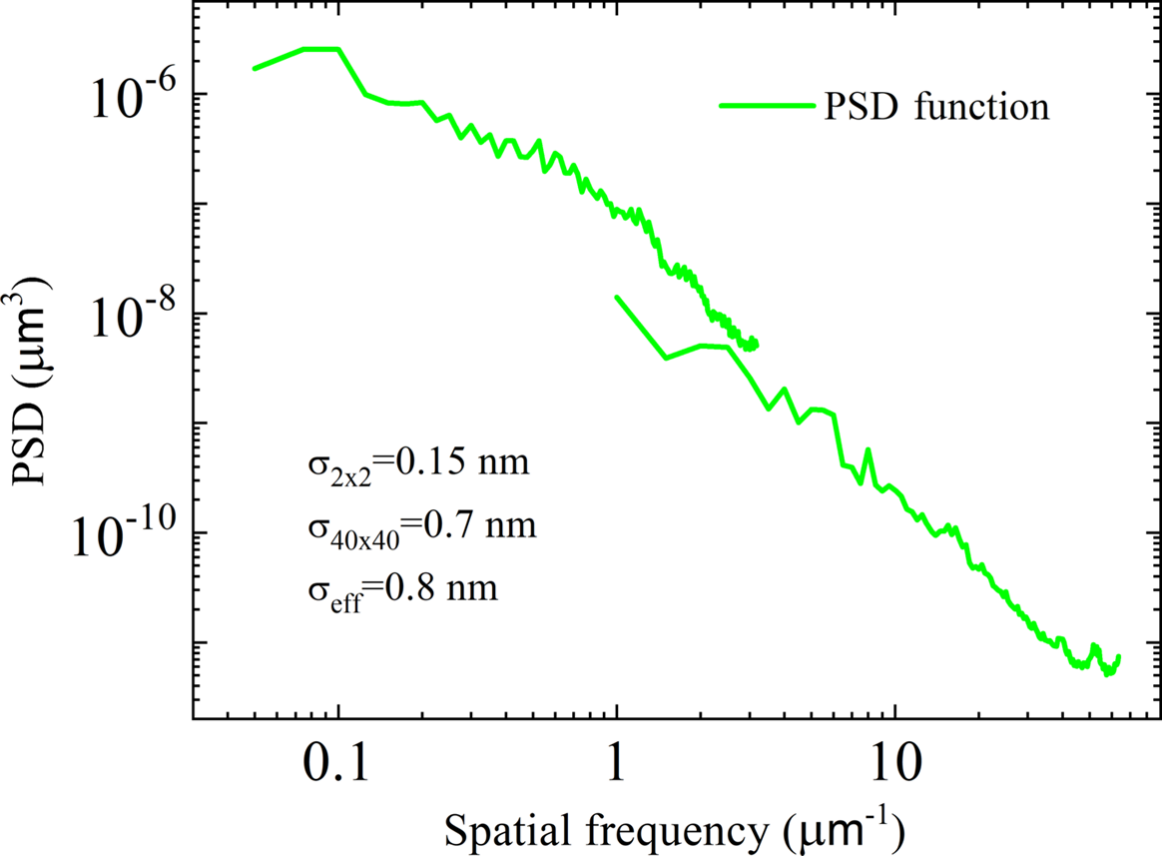
PSD functions of the surface roughness of sample SK1 (AFM data). σ_eff_ = 0.8 nm, σ_2×2_ = 0.15 nm and σ_40×40_ = 0.7 nm.

Fig. 3 ▸ shows the PSD function of the surface roughness of sample SK1, reconstructed from AFM measurements. As can be seen, PSD functions reconstructed from AFM frames of different sizes display a gap, which is explained by an increase in the roughness value due to scratches getting into the frame when its size is increased. The integral value of the effective surface roughness was σ_eff_ = 0.8 nm over the entire range of spatial frequencies ν ∈ [2.5 × 10^−2^ to 6.3 × 10^1^ µm^−1^]. The main contribution to the roughness value is due to the presence of a large number of deep (depth ≃ 10 nm) scratches, whereas the areas between the scratches (frames 2 µm × 2 µm) show good surface smoothness (σ_2×2_ ≃ 0.1 nm).

**Figure 4:**
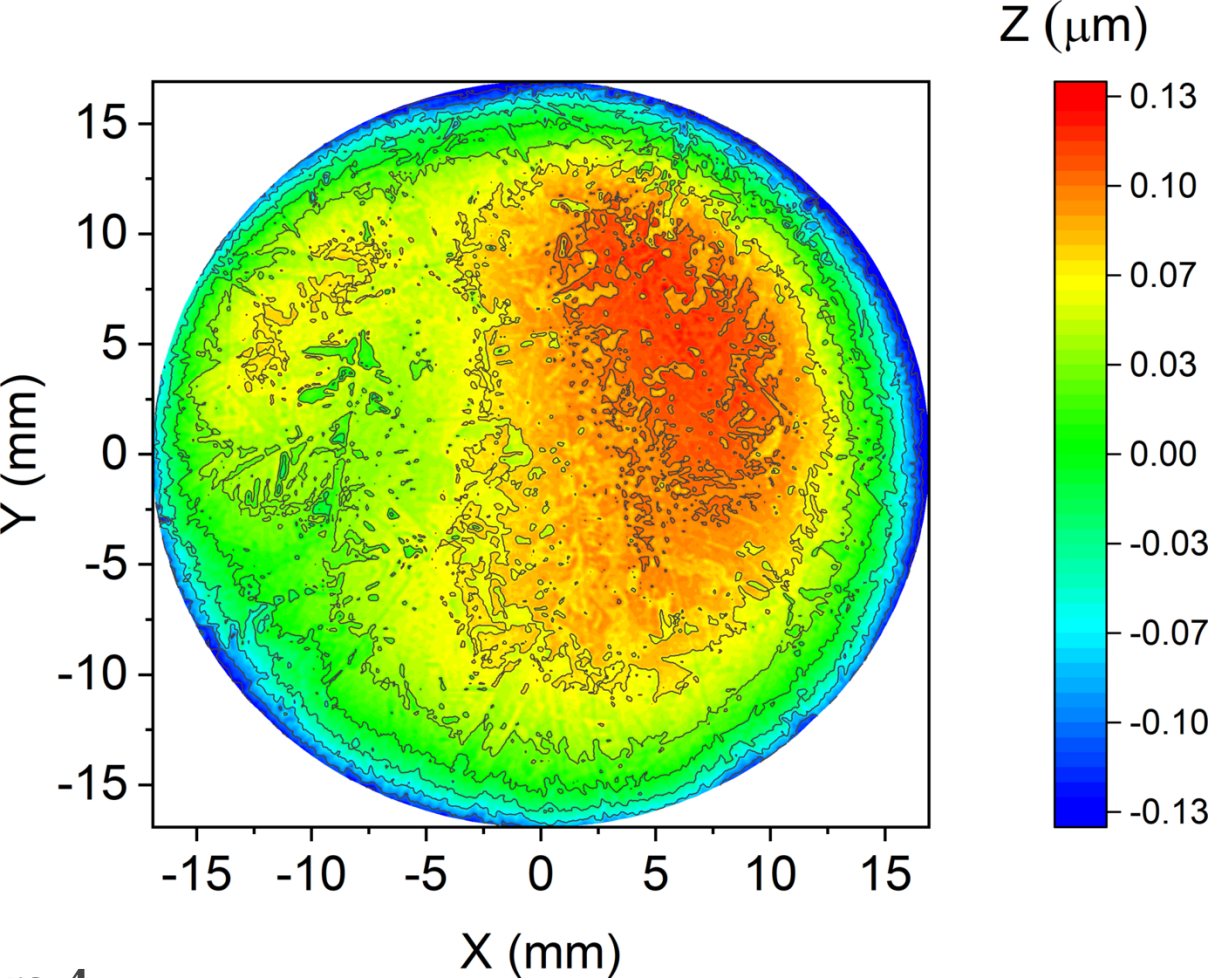
A map of the surface of sample SK2 (90%): PV_90%_ = 232 nm, r.m.s._90%_ = 50.4 nm.

Measurements of the surface shape showed the following: the maximum height span was more than 0.75 µm, with a standard deviation of the surface shape of more than 100 nm. The maximum unflatness is observed at the edges of the sample, while in the central region (90% of the radius) the surface characteristics are much better (Fig. 4 ▸).

The spreads of parameters for both samples were σ_eff_ = 0.8–1.0 nm, PV_90%_ = 230–250 nm and r.m.s._90%_ = 50–60 nm. Such spreads of the roughness and flatness parameters of the surface indicate a proven technology that enables us to obtain identical surfaces with the parameters indicated above.

### 2.3. Thermophysical properties of the DSCC Skeleton^®^

**Figure 5:**
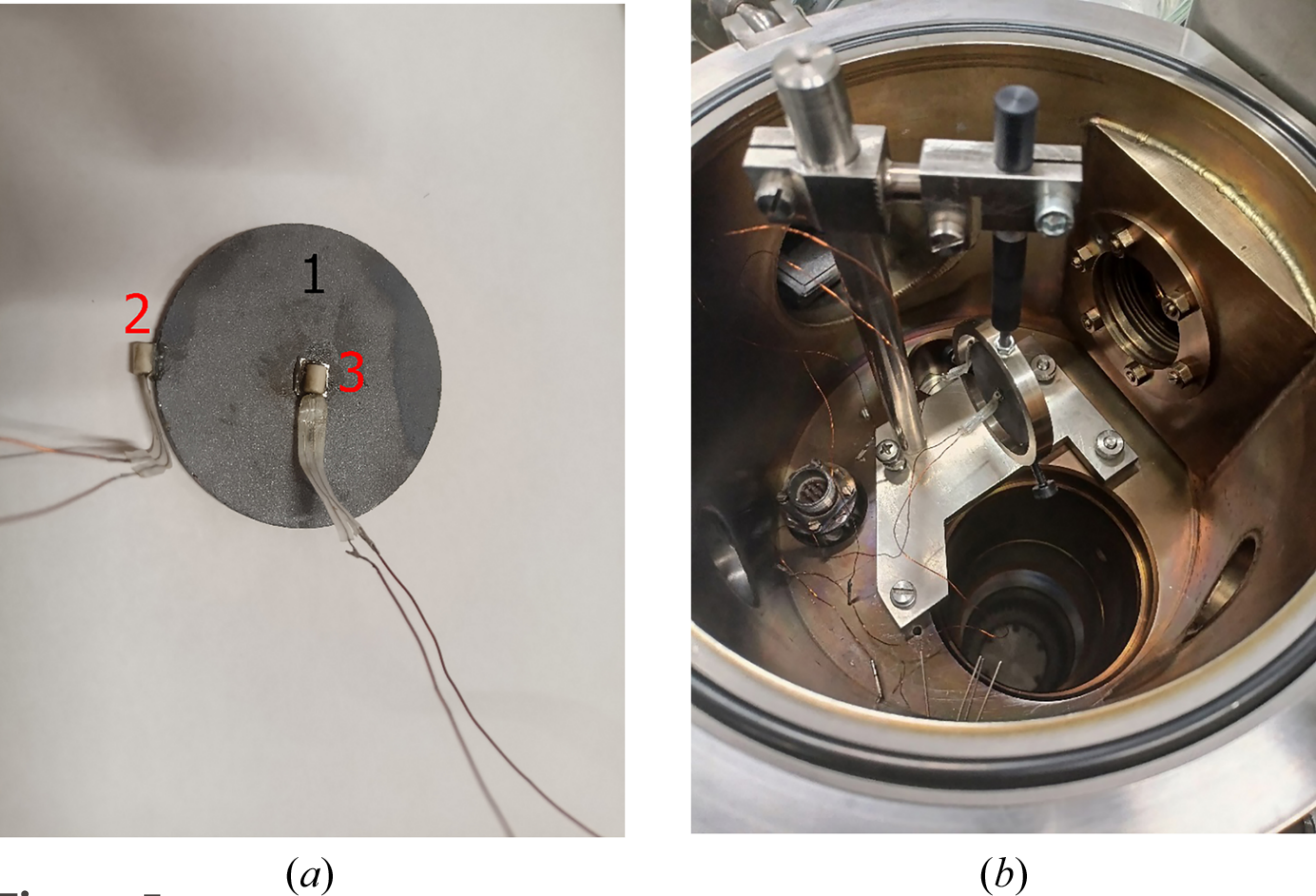
A photograph of sample SK2, (*a*) with the glued sensors and (*b*) in the vacuum chamber. The numbered labels denote 1 – sample SK2, and 2 and 3 – thermoresistive sensors.

The heat capacity and thermal conductivity of Skeleton^®^ were studied in comparison with monocrystalline silicon in our experiments on laser heating the samples in a vacuum, with the temperature being controlled by thermoresistive sensors. A CO_2_ laser was used, with a power of 8 W and a beam size of 5 mm. The samples were mounted using nylon clamps in order to minimize heat sinking to the metal holder. The studies were conducted in a vacuum to exclude convection cooling. Thermo­resistive sensors were glued with heat-conducting glue at the center of the back faces and on the edges of the samples. Photos of sample SK2 with glued sensors and mounted on a holder in the vacuum chamber are shown in Fig. 5 ▸.

The heat capacity and thermal conductivity were estimated from an analysis of the temporal dependence of the temperature and of the temperature difference between the center (heating area) and the boundary of the sample.

The heat capacity was determined at the initial stage of heating using the following ratio:
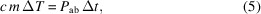
where *c* is the specific heat capacity (J K^−1^ g^−1^), *m* is the mass of the sample, Δ*T* is the temperature increment over the time interval Δ*t* and *P*_ab_ is the absorbed power.

To determine the thermal conductivity, equation (6) was solved:
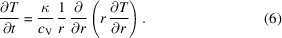
Here κ is the coefficient of thermal conductivity and *c*_V_ is the heat capacity of a unit volume.

The temperature distribution over a thin thermally insulated disk of radius *R* and thickness *D*, locally heated at the center, was modeled by solving equation (6) for a disk with a hole of radius *R*_0_ (*R*_0_
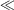
*R*), setting the zero radial derivative of the temperature at *r* = *R*. At the same time, at the boundary of the hole (*r* = *R*_0_), the value of the temperature derivative along the radius *S*_0_ is determined by the power *P* of the heating source (located outside the solution area in the hole area). With the establishment of a stationary distribution of the radial derivative of the temperature, the solution of equation (6) approaches the expression
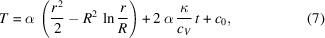
where α = 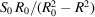, *S*_0_ = −*P*/(κ2π*R*_0_*D*) and *c*_0_ is a constant that depends on the initial conditions. From (7) we can find the temperature difference between the points *r* = *R*_0_ and *r* = *R*:
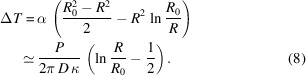
In the experiment, IR laser radiation was injected into the vacuum chamber through a ZnSe window. Previously, the reflection and transmission coefficients of the IR radiation had been measured in the geometry of the experiment for both samples to account accurately for the absorbed power. The results of measurements of the reflection and transmission coefficients are presented in Table 1 ▸. The results of measurements at an angle of 20° to the normal were subsequently used to study any thermally induced deformation of the samples (Section 2.4). The geometric dimensions of the samples are also presented in Table 1 ▸. The absorbed powers for the samples Si1 and SK1 were 2.1 W and 3.7 W, respectively.

**Figure 6:**
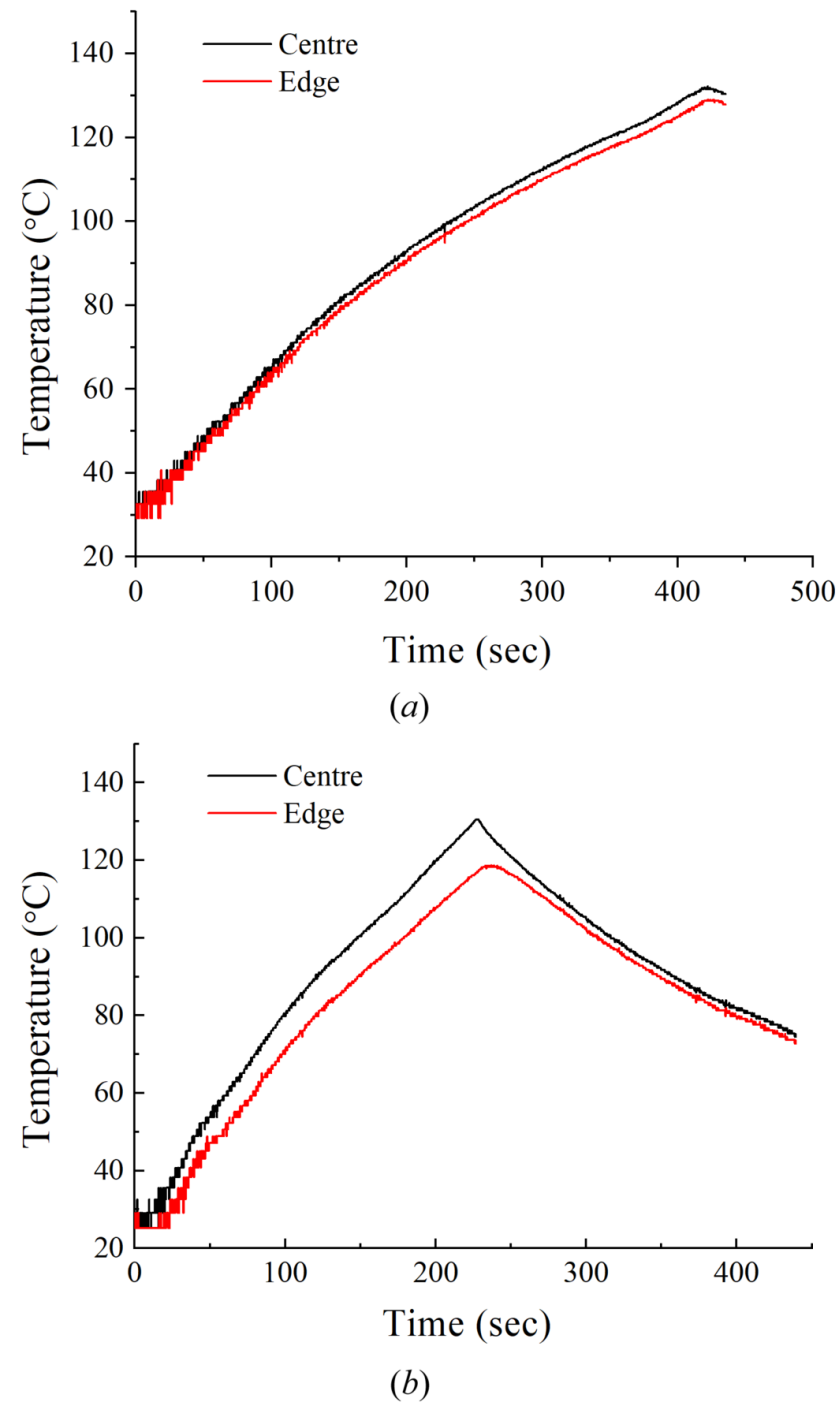
The temporal dependence of temperature, (*a*) for the DSCC Skeleton^®^ (SK1) and (*b*) for silicon (Si1). The black curve is the center of the sample and the red curve is the edge of the sample.

The temporal dependencies of temperature for samples Si1 and SK1, plotted from the data of oscillographic measurements of the resistance of the sensors, are shown in Fig. 6 ▸.

Let us turn to the readings taken from the thermal sensors during heating of a silicon sample [Fig. 6 ▸(*b*)]. During heating, the rate of the rise in temperature of the sample slows down, which is associated with both the increase in specific heat capacity that occurs for silicon and the increase in radiative heat loss. The temperature difference between the center and the edge quickly reaches a value of about 10°C, but towards the end of heating it increases noticeably. This also shows, firstly, a decrease in the thermal conductivity of silicon with increasing temperature, and, secondly, an increase in heat loss due to thermal radiation. Radiation losses near the edge of the disk should be somewhat more efficient due to the contribution of the cylindrical surface that bounds the disk along its outer radius.

The sample of Skeleton^®^ behaves similarly when heated. However, the heating and cooling of this sample are slower due to the greater heat capacity, and the temperature difference between the center and the edge of the sample is noticeably smaller, which is a result of the higher thermal conductivity. During the whole sweep time of the oscilloscope, the Skeleton^®^ sample heats up to a temperature of about 130°C, while the silicon sample heats up to this temperature much faster, in about half the time. When analyzing the experimental data in accordance with the model used, it was discovered that the value of the thermal conductivity of the Skeleton^®^ sample (about 5 W cm^−1^ K^−1^) was almost four times higher than that of the sample of single-crystal silicon (1.3 W cm^−1^ K^−1^). The specific heat capacity of silicon turned out to be 0.9 J K^−1^ g^−1^, which is close to the tabulated value. For Skeleton^®^, the specific heat capacity is much higher and amounts to 1.2 J K^−1^ g^−1^. The noticeably higher thermal conductivity is due to the composition of Skeleton^®^, which includes silicon carbide with a high thermal conductivity and diamond grains with a very high thermal conductivity.

**Figure 7:**
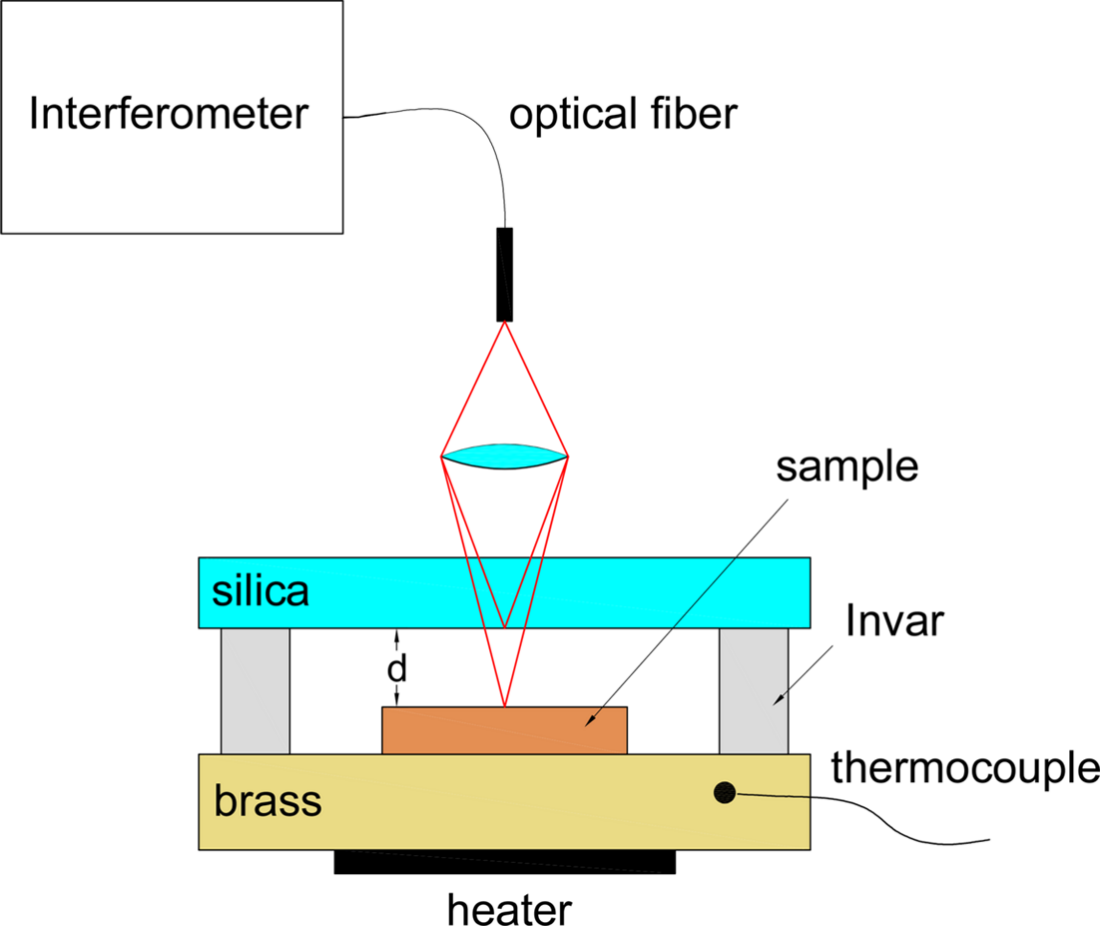
The setup for determining the coefficient of linear thermal expansion.

The coefficient of linear thermal expansion (CLTE) was determined using a fiber interferometer according to the scheme shown in Fig. 7 ▸. The interferometer and the measurement technique are described in detail by Yurasov *et al.* (2015 ▸) and Volkov *et al.* (2015 ▸).

The sample was mounted on a heating panel with a built-in temperature sensor. A fiber interferometer sensor fixed on a thin (0.5 mm) silica plate was mounted above the test sample on Invar 36H racks, with a height of *D*^inv^ = 8.9 mm. The CLTE of Invar is α_inv_ = 1.2 × 10^−6^ K^−1^ in the temperature range 0–100°C. Thus, knowing the CLTE of the Invar racks and controlling the gap between the upper surface of the sample and the lower surface of the silica plate cover, it is possible to determine the CLTE of the sample under study. The change in the gap between the sample and the silica plate can be determined as follows:
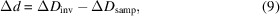
where Δ*d* is the change in the gap between the sample and the silica plate, Δ*D*^inv^ is the change in the height of the Invar racks and Δ*D*^samp^ is the change in the sample thickness.

A series of experiments were carried out. The heating panel was heated from room temperature (26°C) to 70°C (*i.e.* heating by Δ*T* = 44°C). This led to the expansion of the Invar columns by Δ*D*^inv^ = α_inv_Δ*TD*^inv^ = 1.2 × 10^−6^ × 44 × 8.9 × 10^6^ = 470 nm.

To control the method, samples of Si (0.6 and 8.4 mm thick plates) and of fused silica glass (5 mm thick plate) with known CLTE parameters (α_Si_ = 2.8 × 10^−6^ K^−1^ and α_SiO2_ = 5.5 × 10^−7^ K^−1^) were studied. The measurement results are presented in Table 2 ▸. Positive values indicate an increase in the gap Δ*d*, and, conversely, negative values denote a decrease. In the case of the ‘thin’ silicon plate and the silica glass plate, the measured gap increases when heated, which is due to the fact that the thermal expansion of the Invar columns is greater than that of the samples. In the case of silicon, this is due to the small thickness of the plate (0.6 mm) and in the case of the silica plate it is due to its lower CLTE.

As can be seen from the table, there is a good correlation between the measured CLTE values of the silica sample and the tabular values, which confirms the adequacy of the method. For crystalline silicon, a slight deviation of about 10% from the tabular value is observed, which is apparently due to the orientation of the sample cut (3° relative to the 〈110〉 orientation). Thus, using this method of interferometric control of the gap between the heated sample and the interferometric sensor, the CLTE value was measured for the Skeleton^®^ samples and was about α_Sk_ = 4.3 × 10^−6^ K^−1^.

### 2.4. Investigation of thermally induced deformation

The experiment used samples of the DSCC Skeleton^®^ and monocrystalline Si of similar thickness (SK1, *D* = 4.4 mm, and Si2, *D* = 4.0 mm). The optical characteristics (transmission and reflection coefficients) of the samples at the wavelength of the CO_2_ laser (λ = 10.6 µm) are given in Table 1 ▸.

**Figure 8:**
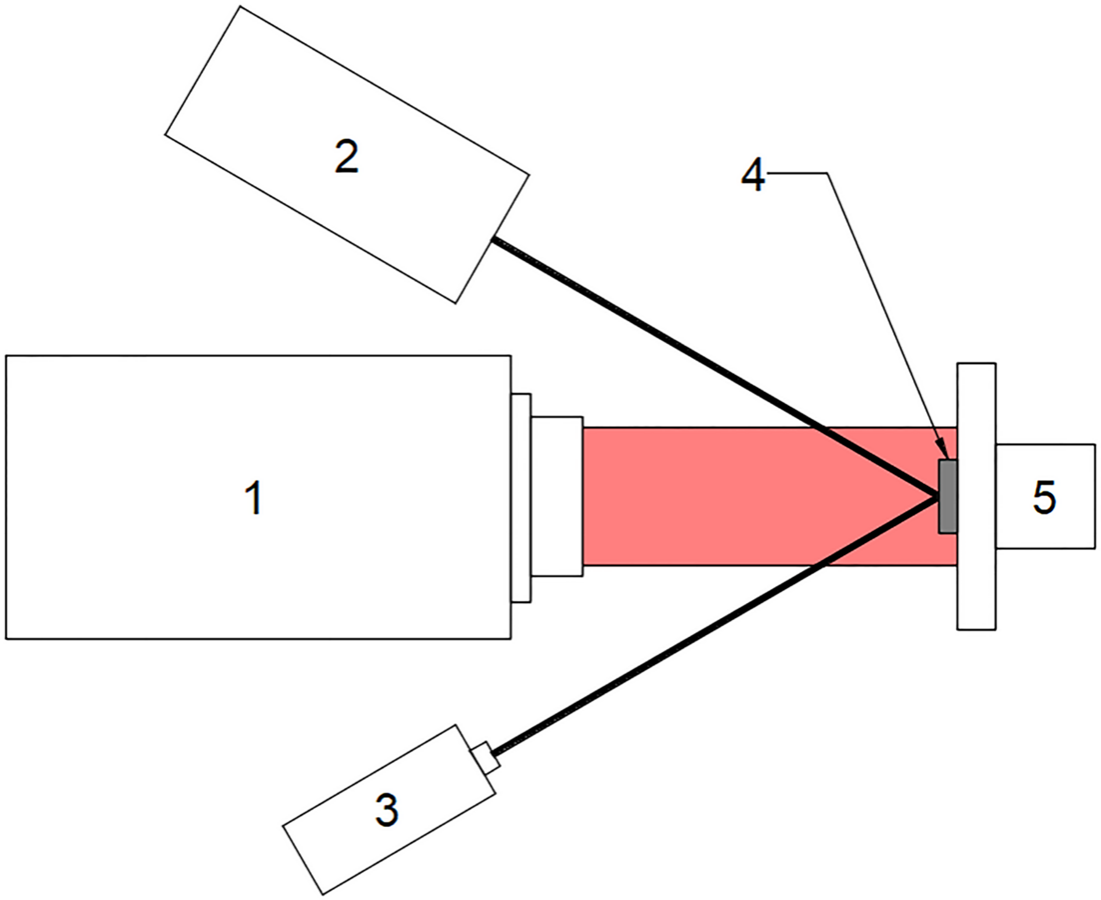
The setup for measurement of thermally induced deformation. The numbered labels denote 1 – the ZYGO VeriFire 4 laser interferometer, 2 – the CO_2_ laser, 3 – the power meter, 4 – the sample and 5 – the 5D table.

The thermally induced deformation of the samples was studied using a Zygo VeriFire 4 laser interferometer. The experimental scheme is presented in Fig. 8 ▸. The sample (labeled 4) was mounted on an adjustable table (5) and the interference pattern was used to adjust the sample plane perpendicular to the optical axis of the interferometer (1). After adjustment, a map of the sample surface was recorded. The sample surface was then locally heated using a CO_2_ laser (2). During the heating process, a series of interferograms were recorded. The deformation map of the sample surface was obtained from the measurement results by subtracting the surface map before heating from the surface map during heating. The stability of the laser operation during the experiment was controlled by the magnitude of the reflected radiation using an IR radiation power meter (3).

**Figure 9:**
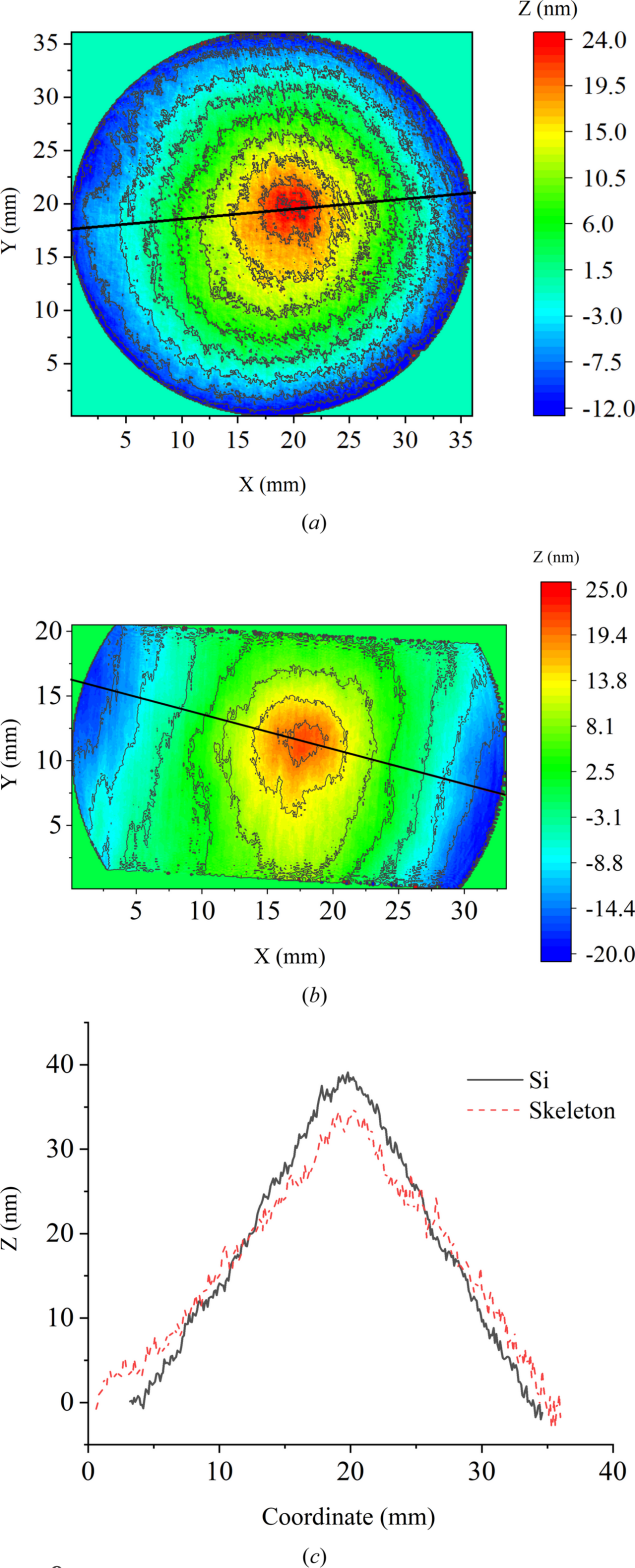
(*a*) and (*b*) Maps of the thermally induced deformation of the surfaces of samples (*a*) SK1, DSCC Skeleton^®^ and (*b*) Si2, monocrystalline silicon. (*c*) Cross sections of the surface profiles of samples SK1 and Si2.

As follows from the measurements presented in Table 1 ▸, the power absorbed by the experimental samples was: for SK1 *P* = 6.0 W and for Si2 *P* = 3.3 W. The resulting maps of the deformation of the surface of the silicon and Skeleton^®^ samples are shown in Fig. 9 ▸. The deformation of both samples is similar in magnitude. The ranges of heights according to the series of measurements were: PV_SK_ = 33.0±2.0 nm and PV_Si_ = 38.2±2.0 nm. At first glance, the result looks unexpected. The thermally induced deformations of Skeleton^®^ and Si turned out to be close, while the thermophysical characteristics of Skeleton^®^ are better.

**Figure 10:**
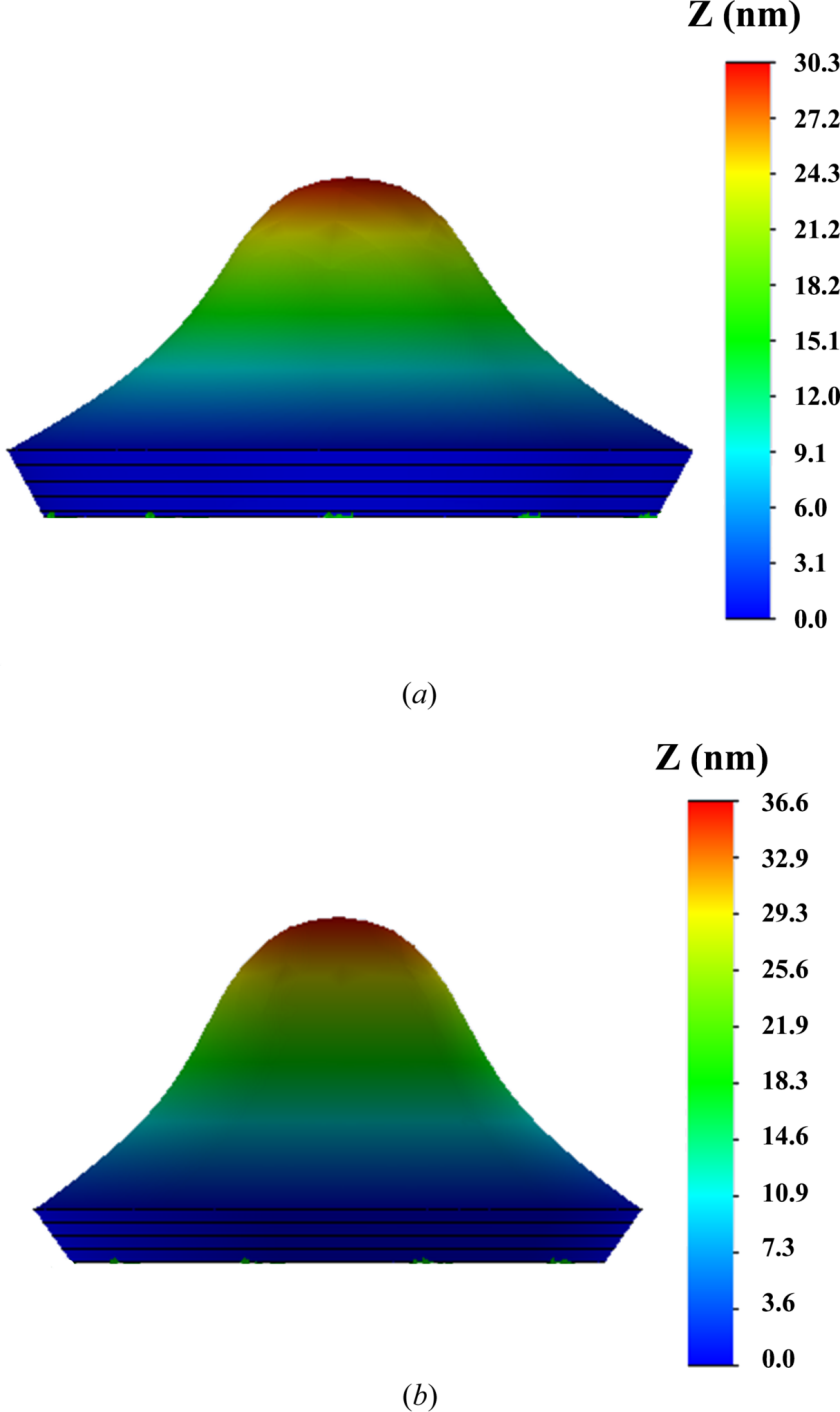
Maps of the thermally induced deformation of the surfaces of samples due to exposure to CO_2_ laser radiation, (*a*) DSCC Skeleton^®^ (PV_Sk_ = 30.3 nm) and (*b*) monocrystalline Si (PV_Si_ = 36.6 nm). The calculation was performed using *SolidWorks* software.

To explain the results of the laser heating experiment, a simulation of thermally induced deformation was carried out using *SolidWorks* software (Dassault Systèmes, https://www.solidworks.com/). As thermophysical characteristics, the tabular data for monocrystalline silicon and the values obtained in this work for the DSCC Skeleton^®^ were included in the calculation. Fig. 10 ▸ shows the simulation results. There is good correspondence between the measured and calculated profiles of thermally induced deformations in both samples, which indicates the reliability of the measured thermophysical constants of Skeleton^®^.

The physical reason for the seeming contradiction is that, firstly, a practically two times greater amount of energy was absorbed by the Skeleton^®^ sample – 6.0 W compared to 3.3 W for Si – and, secondly, the heat is absorbed mainly near the surface of the DSCC Skeleton^®^, whereas in monocrystalline Si, the absorbed power is distributed throughout the thickness of the plate due to the good transmission of Si in the vicinity of the wavelength of 10.6 µm.

In the case of X-ray irradiation of the samples, both the magnitude and the depth profile of the absorbed power will be close. In this case, it can be expected that the deformation of a mirror on a substrate made from Skeleton^®^ will be significantly smaller. To test this assumption, modeling was performed in *SolidWorks*. The area exposed to X-rays was chosen as a long narrow strip, taking into account the typical application of such mirrors for grazing-angle focusing of synchrotron radiation.

**Figure 11:**
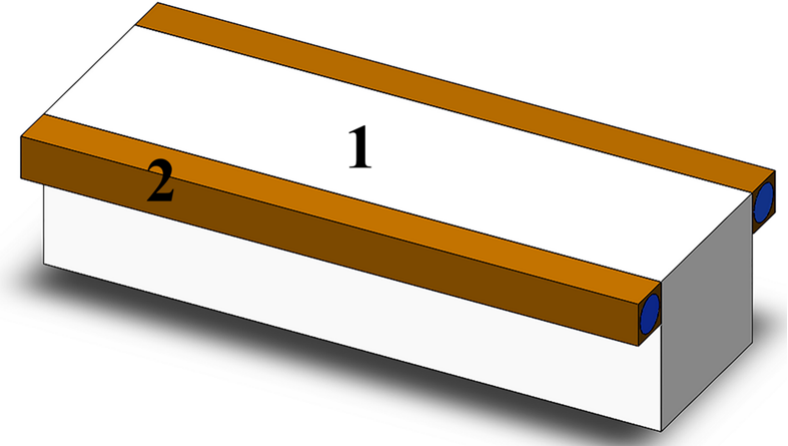
A statement of the problem: a sample with water-cooled radiators. Label 1 indicates the substrate and 2 the water-cooled copper radiators.

The deformation of substrates made of Skeleton^®^, monocrystalline silicon and diamond with dimensions of 200 mm × 25 mm × 25 mm was calculated under the influence of a 200 W heat flux onto an area of 4 mm × 180 mm. The grazing angle of the incoming X-ray beam in the numerical experiment was assumed to be 0.95°. Such conditions are expected on Station 1-1, the ‘Microfocus’ beamline of the Synchrotron Radiation Facility Siberian Circular Photon Source (SRF SKIF, Novosibirsk, Russia). A value of 200 W is the typical power falling on the primary optical element. Calculations with this power value were carried out (Brumund *et al.*, 2021 ▸) for the ESRF synchrotron, which, after modernization, belongs to the fourth generation. Heat removal is realized via liquid cooling in the upper part of the substrate with a coefficient of 3 kW m^−2^ K^−1^. Due to the symmetry, let us consider the thermal deformation of half of the mirror (see Fig. 11 ▸).

**Figure 12:**
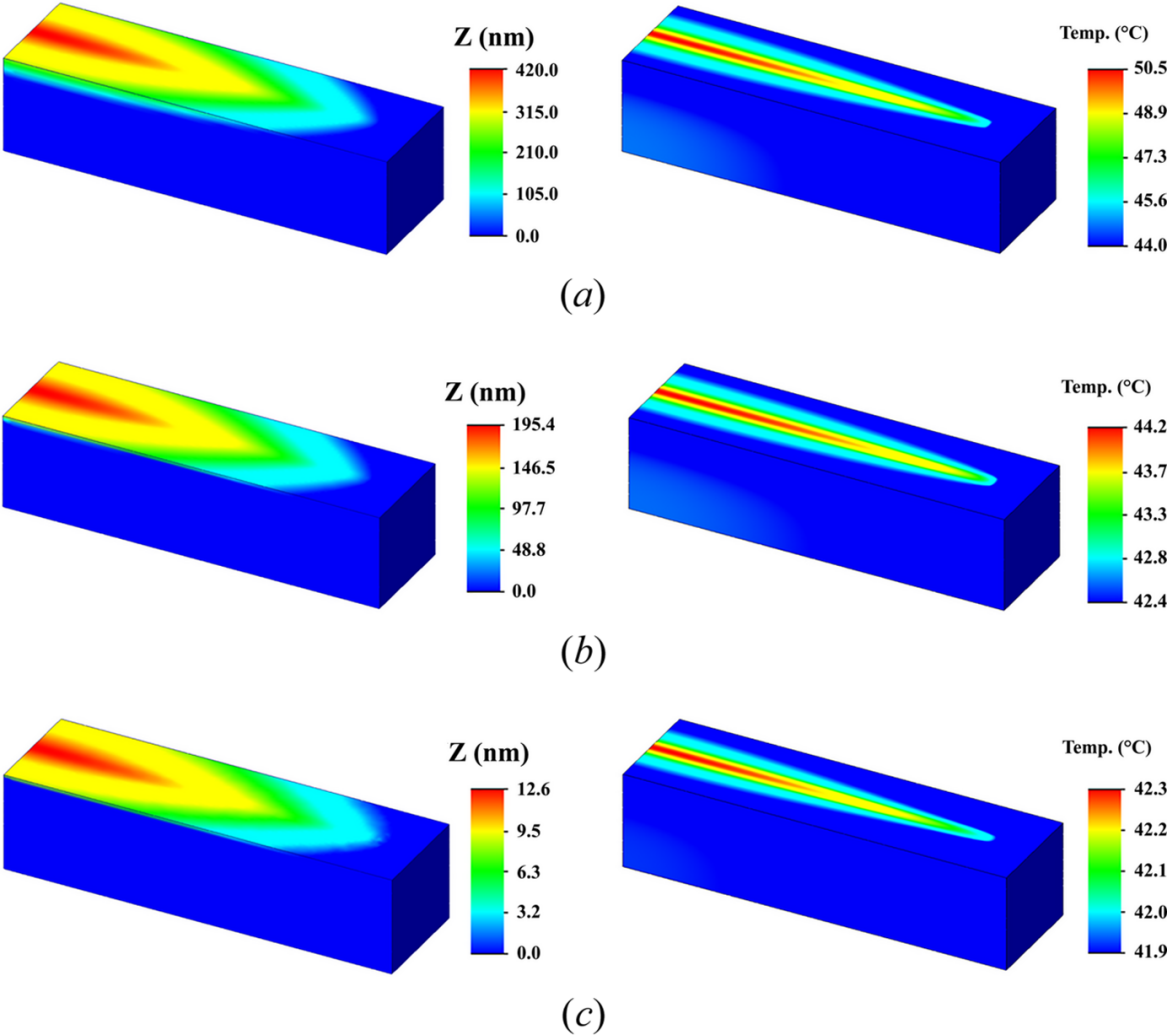
Maps of thermally induced deformation and temperature distribution for (*a*) single-crystal silicon, (*b*) the DSCC Skeleton^®^ and (*c*) single-crystal diamond. Heating with hard (*E* = 20 keV) X-ray synchrotron radiation (calculation using *SolidWorks* software).

**Figure 13:**
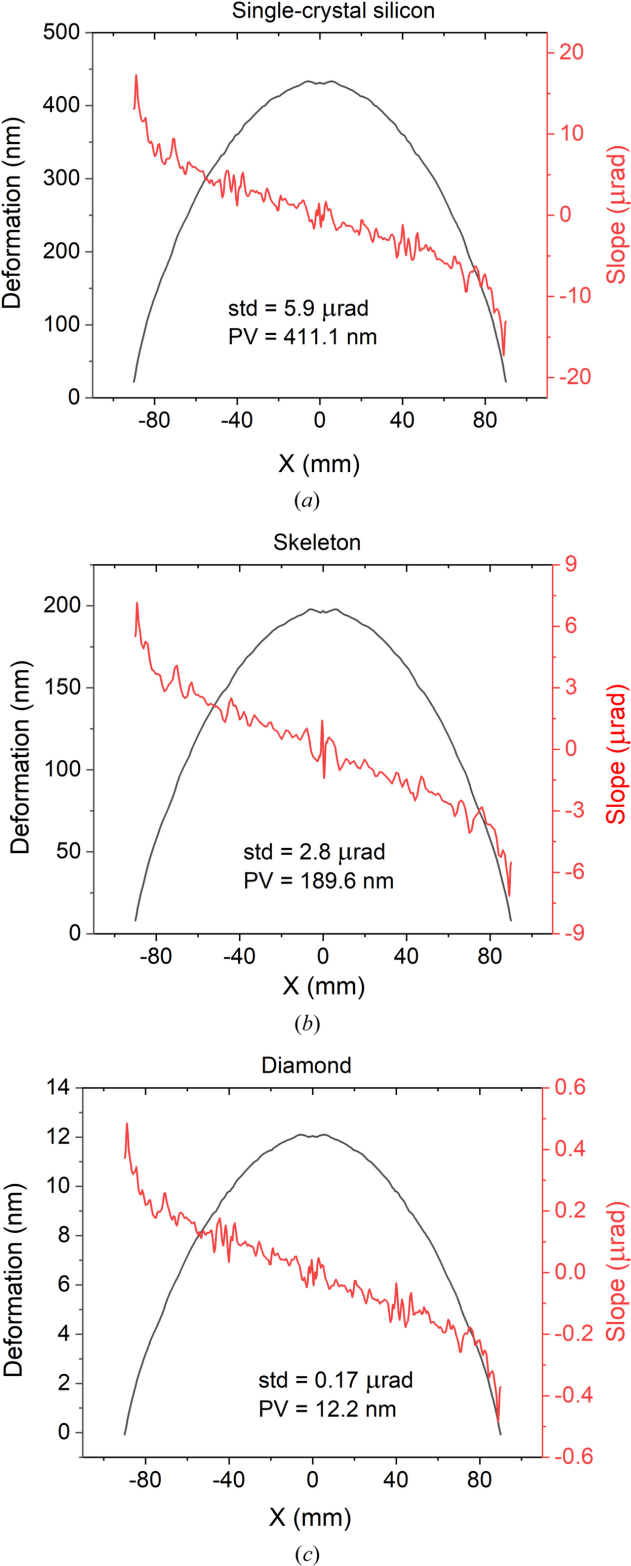
Cross-sections of maps of thermally induced deformation under exposure to hard X-ray radiation: (*a*) monocrystalline silicon (PV_Si_ = 411.1 nm, std = 5.9 µrad), (*b*) DSCC Skeleton^®^ (PV_Sk_ = 189.6 nm, std = 2.8 µrad) and (*c*) monocrystalline diamond (PV_D_ = 12.2 nm, std = 0.17 µrad) (calculation in *SolidWorks *software). The abbreviation ‘std’ (standard deviation) means the root-mean-square of slope deviation from the plane.

The orange bars in the upper part of the sample are water-cooled copper radiators, *i.e.* there is an area in which convective heat transfer occurs. Thus, forced cooling with water at a temperature of 22°C was considered in the simulation. Fig. 12 ▸ shows 3D models of the calculated thermally induced deformations and Fig. 13 ▸ shows their axial sections.

It can be seen that, with increasing thermal conductivity of the materials, the temperature difference within the heated area decreases (silicon PV = 5.5°C; Skeleton^®^ PV = 1.8°C; diamond PV = 0.4°C), which leads to smaller deformation of the substrate surface and, accordingly, smaller deviation of the reflected rays from the calculated direction. The magnitude of the thermally induced deformation on the Skeleton^®^ substrate is noticeably smaller (by more than two times) than the deformation of the surface of the single-crystal silicon sample. However, compared with single-crystal diamond, the deformation is more than 15 times greater. Thus, the DSCC Skeleton^®^ is significantly superior to silicon in terms of its thermophysical characteristics, but is much inferior to single-crystal diamond. To date, the production of substrates with dimensions of several tens of centimetres from single-crystal diamond seems impossible, and therefore the potential for using the DSCC Skeleton^®^ as a mirror substrate material for powerful X-ray sources is obvious.

## 3. Conclusion

The diamond-silicon carbide composite Skeleton^®^, with a thin coating of polycrystalline silicon, was proposed as a material for use in manufacturing substrates for X-ray mirrors operating under high-intensity radiation beams. It has been shown that the polycrystalline silicon coating makes it possible to form a surface with parameters acceptable for subsequent ion-beam polishing and shape correction. The micro-roughness (σ_2×2_) turned out to be at a level of 0.15 nm, which corresponds to the values obtained with standard substrates for multilayer X-ray mirrors made of such materials as fused silica, sitall, ULE and Zerodur (Keller *et al.*, 2009 ▸; Chkhalo *et al.*, 2014 ▸; Kurashima *et al.*, 2008 ▸; Liao *et al.*, 2014 ▸). The rather large values of middle-frequency roughness observed in the experiment are associated with the presence of scratches and with the use of a standard polishing method. We believe that an improved process of chemical-mechanical polishing using suspensions of cerium oxide with a grain size of 0.05–0.1 µm, which our team has developed in recent years, will permit the achievement of ångström levels of roughness while eliminating scratches (Chkhalo *et al.*, 2022 ▸).

The thermophysical constants of Skeleton^®^ have been determined for the first time. The thermal conductivity and heat capacity turned out to be higher than those of monocrystalline silicon and amounted to 5.0 W cm^−1^ K^−1^ and 1.2 J K^−1^ g^−1^, respectively. The coefficient of linear thermal expansion also turned out to be higher than that of silicon: 4.3 × 10^−6^ K^−1^ for Skeleton^®^ versus 3.1 × 10^−6^ K^−1^ for Si.

The reliability of the experimentally obtained constants has been confirmed in two ways. Firstly, single-crystal silicon samples were examined by the same methods and under the same conditions, and the measurement results coincided well with the tabular data. Secondly, an experiment was conducted to measure the thermally induced deformation of Si and Skeleton^®^ samples under exposure to CO_2_ laser radiation with a power of 8 W. The results of the experiment and its modeling using the thermophysical constants that we have obtained coincided with good accuracy.

Calculation in the *SolidWorks* software for the case of heating with X-ray radiation showed that the thermally induced deformation of the surface of a sample made from Skeleton^®^ with a technological coating of polycrystalline silicon will be almost three times less than that of monocrystalline silicon. In general, the thermophysical characteristics of Skeleton^®^ lie between those of monocrystalline silicon and diamond.

Thus, the research we have conducted indicates that potentially the new material, the DSCC Skeleton^®^, can be used as an alternative to monocrystalline silicon for X-ray mirror substrates when used in conditions of high-intensity electromagnetic radiation beams, such as free-electron lasers, third+ and fourth-generation synchrotrons, and ultra-power laser systems. The material has good polishability, a low cost of components (micro- or nano-powders) and production (technology for creating ceramic materials), and no restrictions on the shape and size of manufactured substrates.

For practical applications, the long-term stability of the substrate material under the influence of powerful X-ray beams is important. Unfortunately, there is no such literature data on this material yet. However, the high-temperature manufacturing process of Skeleton^®^, the proven long-term stability of the components (diamond, silicon and silicon carbide) of which it consists, the presence of electrical conductivity, and the absence of any organics allow us to hope for the high dimensional and temporal stability of this mater­ial.

Further study of the DSCC Skeleton^®^ with a thin technological coating of polycrystalline silicon involves obtaining the required level of surface roughness using mechanical and chemical-mechanical polishing methods, studying the effect of ion etching on surface roughness for the purpose of using IBF technology for surface shape correction, and coating the treated surfaces with multilayer X-ray mirrors and studying their X-ray optical characteristics.

## Figures and Tables

**Table 1:** Optical properties of experimental samples at a wavelength of 10.6 µm *D* is the sample thickness, *L* the length of the sample (for Si2 only) and *W* its width. *R*_10.6_ is the reflection coefficient and *T*_10.6_ is the transmission coefficient at a wavelength of 10.6 µm.

Sample	*R*_10.6_(⊥) (%)	*T*_10.6_(⊥) (%)	*R*_10.6_(20°) (%)	*T*_10.6_(20°) (%)
ZnSe (vacuum window)	35.0	65.0	–	–
Si1 (*D* = 3 mm, ∅ = 46 mm)	29.0	30.2	–	–
Si2 (*D* = 4 mm, *L* = 48 mm, *W* = 20 mm)	29.5	27.9	28.8	29.6
SK1 (*D* = 4.4 mm, ∅ = 40 mm)	28.9	0	25.3	0

**Table 2:** CLTE measurement results

	26°C → 70°C	70°C → 26°C	26°C → 70°C	70°C → 26°C		
Sample	Δ*d* (nm)	Δ*d* (nm)	Δ*d* (nm)	Δ*d* (nm)	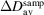 (nm)	α (K^−1^)
Si3 (*D* = 0.6 mm)	385	−397	393	−388	79.2±4.0	(3.0±0.16) × 10^−6^
Si4 (*D* = 8.4 mm)	−690	702	−670	718	1165.0±33.8	(3.1±0.16) × 10^−6^
SK1 (*D* = 4.4 mm)	−358	347	−379	328	823.0±46.9	(4.2±0.24) × 10^−6^
SK2 (*D* = 4.4 mm)	−378	396	−368	375	849.2±45.0	(4.4±0.24) × 10^−6^
SiO_2_ (*D* = 5.0 mm)	344	−330	350	−366	347.5±20.2	(5.5±0.33) × 10^−7^

## Data Availability

The data that support the findings of this study are available from the corresponding author upon reasonable request.

## References

[bb1] Andreev, S. S., Bibishkin, M. S., Chkhalo, N. I., Kluenkov, E. B., Prokhorov, K. A., Salashchenko, N. N., Zorina, M. V., Schafers, F. & Shmaenok, L. A. (2003). *J. Synchrotron Rad.* **10**, 358–360. 10.1107/s0909049503015255 12944620

[bb2] Assoufid, L. & Graafsma, H. (2017). *MRS Bull.* **42**, 418–423.

[bb3] Barysheva, M. M., Chkhalo, N. I., Drozdov, M. N., Mikhailenko, M. S., Pestov, A. E., Salashchenko, N. N., Vainer, Yu. A., Yunin, P. A. & Zorina, M. V. (2019). *J. X-ray Sci. Technol.* **27**, 857–870. 10.3233/XST-190495 31282467

[bb5] Belure, A. R., Biswas, A. K., Raghunathan, D., Bhartiya, R. S., Rai, S. K., Pawade, R. S., Kamath, M. P. & Benerji, N. S. (2019). *International Conference on Precision, Meso, Micro and Nano Engineering (COPEN2019)*, 12–14 December 2019, Indore, India.

[bb4] Belure, A. R., Biswas, A. K., Raghunathan, D., Bhartiya, R. S., Singh, R., Rai, S. K., Pawade, R. S., Kamath, M. P. & Benerji, N. S. (2020). *Mater. Today Proc.* **26**, 2260–2264.

[bb6] Born, M. & Wolf, E. (1999). *Principles of Optics*, p. 528. Cambridge University Press.

[bb7] Brumund, P., Reyes-Herrera, J., Detlefs, C., Morawe, C., Sanchez del Rio, M. & Chumakov, A. I. (2021). *J. Synchrotron Rad.* **28**, 91–103. 10.1107/S1600577520014009 33399557

[bb8] Chkhalo, N, I., Akhsakhalyan, A. A., Vainer, Yu. A., Zorina, M. V., Pestov, A. E., Svechnikov, M. V., Toropov, M. N., Kumar, N. & Tokunov, Yu. M. (2022). *Tech. Phys.* **92**, 2146.

[bb9] Chkhalo, N. I., Churin, S. A., Pestov, A. E., Salashchenko, N. N., Vainer, Y. A. & Zorina, M. V. (2014). *Opt. Express*, **22**, 20094–20106. 10.1364/OE.22.020094 25321219

[bb10] Chkhalo, N. I., Malyshev, I. V., Pestov, A. E., Polkovnikov, V. N., Salashchenko, N. N. & Toropov, M. N. (2020). *Phys-Usp.* **63**, 67–82.

[bb11] Chkhalo, N. I., Salashchenko, N. N. & Zorina, M. V. (2015). *Rev. Sci. Instrum.* **86**, 016102. 10.1063/1.4905336 25638129

[bb12] Chkhalo, N. I., Zorina, M. V., Malyshev, I. V., Pestov, A. E., Pol­kovnikov, V. N., Salashchenko, N. N., Kazakov, D. S., Mil’kov, A. V. & Strulya, I. L. (2019). *Tech. Phys.* **64**, 1596–1601.

[bb13] DiGennaro, R., Gee, B., Guigli, J., Hogrefe, H., Howells, M. & Rarback, H. (1988). *Nucl. Instrum. Methods Phys. Res. A*, **266**, 498–506.

[bb14] Kataev, S., Sidorov, V. & Gordeev, S. (2011). *Electronics STB*, **3**, 60–64.

[bb23] Keller, A., Facsko, S. & Möller, W. (2009). *J. Phys. Condens. Matter*, **21**, 495305. 10.1088/0953-8984/21/49/495305 21836193

[bb15] Khounsary, A., Fernandez, P., Assoufid, L., Mills, D., Walters, D., Schwartz, J. & Robichaud, J. (2002). *Rev. Sci. Instrum.* **73**, 1537–1540.

[bb24] Kurashima, Y., Miyachi, S., Miyamoto, I., Ando, M. & Numata, A. (2008). *Microelectron. Eng.* **85**, 1193–1196.

[bb16] Liao, W., Dai, Y., Xie, X. & Zhou, L. (2014). *Opt. Express*, **22**, 200281. 10.1364/OE.22.000377 24514998

[bb17] Morawe, Ch., Barrett, R., Friedrich, K., Klünder, R. & Vivo, A. (2013). *J. Phys. Conf. Ser.* **425**, 052027.

[bb18] Shvyd’ko, Y., Terentyev, S., Blank, V. & Kolodziej, T. (2021). *J. Synchrotron Rad.* **28**, 1720–1728. 10.1107/S1600577521007943 34738925

[bb19] Ulmeanu, M., Serghei, A., Mihailescu, I. N., Budau, P. & Enachescu, M. (2000). *Appl. Surf. Sci.* **165**, 109–115.

[bb20] Volkov, P. V., Goryunov, A. V., Lukyanov, A. Yu., Okhapkin, A. I., Tertyshnik, A. D., Travkin, V. V. & Yunin, P. A. (2015). *Appl. Phys. Lett.* **107**, 111601.

[bb21] Wang, Z., Wu, L., Fang, Y., Dun, A., Zhao, J., Xu, X. & Zhu, X. (2022). *Micromachines*, **3**, 318. 10.3390/mi13020318 PMC8877455 35208442

[bb22] Yurasov, D. V., Luk’yanov, A. Yu., Volkov, P. V., Goryunov, A. V., Tertyshnik, A. D., Drozdov, M. N. & Novikov, A. V. (2015). *J. Cryst. Growth*, **413**, 42–45.

